# The evolving deceased donor kidney transplantation programme in Sri Lanka: current landscape, constraints, and next steps

**DOI:** 10.3389/frtra.2026.1811682

**Published:** 2026-05-26

**Authors:** Anura Hewageegana

**Affiliations:** Department of Nephrology, National Hospital of Sri Lanka, Colombo, Sri Lanka

**Keywords:** deceased donor kidney transplantation, health systems implementation, immunobiology in transplantation, kidney allocation system (KAS), resource limited settings, transplant governance, transplant registry and auditability

## Abstract

Deceased donor kidney transplantation (DDKT) is an essential strategy to improve access to kidney replacement therapy in low- and middle-income countries (LMICs), where haemodialysis capacity remains insufficient and long-term peritoneal dialysis is under-utilised. In Sri Lanka, deceased donation has evolved from sporadic, centre-driven activity towards a more structured national programme with clearer policy direction, defined retrieval zones, and gradually standardised clinical workflows. However, safe expansion of DDKT requires more than policy development. It depends on practical investment in implementation systems, including protected transplant coordination roles, secure and reliable information platforms, and timely access to quality-assured immunobiology services. At present, consistent national execution is constrained by the incomplete availability of three key enabling components: (i) a real-time national transplant waitlist with accurate candidate status, (ii) an end-to-end organ offer and acceptance workflow that produces a permanent audit trail, and (iii) time-critical immunobiology capacity to support compatibility assessment for each deceased donor offer. In this context, zone-based coordination with standardised but largely manual allocation processes continues to function as a pragmatic transitional safety framework. Nevertheless, this model limits reproducibility, equity monitoring, and systematic programme learning. This Perspective summarises the current policy and operational environment, identifies predictable bottlenecks across the donation-to-transplant pathway, and proposes a staged systems roadmap that prioritises safety and auditability before automation. Key priorities include structured manual allocation processes with mandatory documentation and key performance indicator (KPI) reporting, development of a minimum viable national registry with a secure offer workflow, and phased strengthening of immunobiology services to enable gradually automated and quality-assured national allocation. The Sri Lankan experience offers practical and potentially transferable lessons for LMIC transplant programmes seeking to scale deceased donor transplantation while maintaining patient safety, equity, and public trust.

## Introduction

Kidney transplantation provides superior survival, better health-related quality of life, and greater long-term cost-effectiveness compared with maintenance dialysis. In addition, the duration of dialysis before transplantation is an important modifiable determinant of both graft survival and patient outcomes (1–4).

In many low- and middle-income countries (LMICs), these recognised benefits are difficult to realise in practice. Dialysis capacity often remains insufficient to meet population needs. Long-term use of peritoneal dialysis is also limited, and access to transplant immunobiology services is uneven. Furthermore, transplant information systems are frequently underdeveloped. Sri Lanka reflects many of these structural realities. Haemodialysis provision continues to fall short of national demand, while long-term peritoneal dialysis remains under-utilised (5, 6).

These constraints make the safe expansion of deceased donor kidney transplantation (DDKT) a clinical and ethical priority. However, programme growth must occur within a framework that ensures patient safety, equity in allocation, and transparency in decision-making.

Over time, Sri Lanka's DDKT programme has evolved from sporadic, centre-led activity towards a more structured national service with clearer policy direction and defined organ retrieval pathways. Instead, the key challenge relates to practical implementation capacity. Several foundational operational enablers required for consistent national allocation remain incompletely established at national level. These include,

(i) A real-time national transplant waitlist with accurate candidate status,
(ii) A secure end-to-end organ offer and acceptance workflow capable of generating a permanent audit trail, and
(iii) Reliable, time-critical immunobiology support for compatibility assessment for each deceased donor offer.

Accordingly, this Policy and Systems Perspective reviews the current operational landscape, identifies the main bottlenecks that constrain performance across the donation-to-transplant pathway, and proposes pragmatic next steps. These recommendations prioritise safety and auditability before automation. Key components include standardised manual allocation processes with mandatory documentation and key performance indicator reporting, development of a minimum viable national registry with a secure offer workflow, and phased strengthening of immunobiology capacity aligned with realistic deceased donor timelines and national service continuity.

## Current landscape and donation pathway

The legal foundation for deceased donor organ transplantation in Sri Lanka is provided by the Transplantation of Human Tissues Act No. 48 of 1987, which established statutory safeguards governing organ retrieval, consent processes, and authorised transplant activity. Subsequent Ministry of Health circulars and national strategic policy documents have progressively operationalised these provisions through structured development of deceased-donor identification pathways, retrieval coordination mechanisms, and allocation governance frameworks.

Organ donation currently operates within an opt-in framework. In the absence of large-scale prospective donor registration systems with reliable verification mechanisms, final authorisation for donation is usually determined through family decision-making at the time a potential donor is identified.

Sri Lanka's kidney transplant programme has evolved over more than four decades, with living-donor transplantation established first and deceased-donor activity developing later, primarily within government-sector transplant centres. The government health sector is estimated to operate approximately 13 active kidney transplant centres across the country. In addition, approximately 10 private hospitals perform kidney transplantation, largely confined to living-donor pathways. National transplant activity is estimated at approximately 350–400 kidney transplants annually, based on aggregated centre-reported programme summaries rather than a harmonised national dataset (7, 8). Sri Lanka's kidney transplant programme has evolved over more than four decades, with living-donor transplantation established first and deceased-donor activity developing later, primarily within government-sector transplant centres. Within this overall activity, deceased donor kidney transplantation (DDKT) accounts for a minority share, contributing approximately 20%–30% of annual transplants; however, a clear upward trajectory is evident, with volumes increasing from fewer than 20 procedures per year in the early 2010s to approximately 90–110 annually in recent years, despite some year-to-year variability.

**Figure 1:**
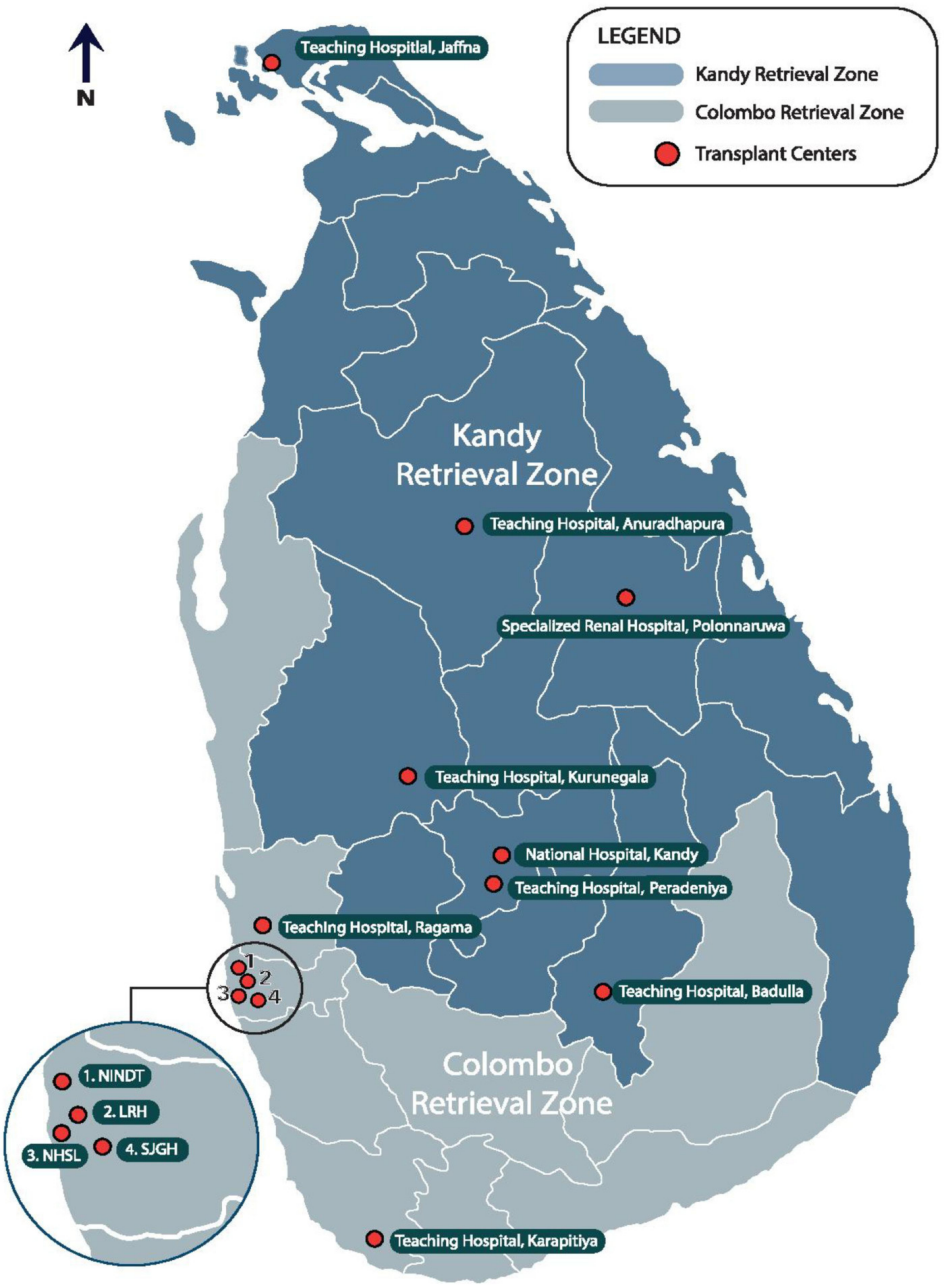
Geographic organization of deceased donor kidney transplantation in Sri Lanka. Schematic map illustrating the current organ retrieval zones and the distribution of major government-sector kidney transplant centers. The programme is organized into two principal retrieval zones: the Colombo retrieval zone and Kandy retrieval zone. Red markers indicate active government sector transplant centers. The inset highlights the Colombo metropolitan cluster, including National Institute of Nephrology, Dialysis and Transplantation (NINDT), Lady Ridgeway Hospital for Children (LRH), National Hospital of Sri Lanka (NHSL), and Sri Jayewardenepura General Hospital (SJGH). The map reflects the current operational structure based on zonal coordination rather a fully implemented national allocation system. NINDT, National Institute of Nephrology, Dialysis and Transplantation; LRH, Lady Ridgeway Hospital for Children; NHSL, National Hospital of Sri Lanka; SJGH, Sri Jayewardenepura General Hospital.

To enhance clarity regarding the geographical and logistical structure of the deceased donor programme in Sri Lanka, we have incorporated a schematic map illustrating the current organ retrieval zones and the distribution of major government-sector transplant centres (Figure 1). It is important to emphasise that, despite the existence of a national allocation algorithm, its routine application remains limited by prevailing logistical, infrastructural, and immunobiological constraints, including the absence of uniformly available real-time immunological testing and integrated allocation infrastructure. The deceased donor programme remains confined to the government sector, while private hospitals predominantly perform living donor transplantation and are not part of the deceased donor allocation pathway. This contextual clarification is essential for accurately interpreting access dynamics and for situating Sri Lanka's model within the broader spectrum of emerging transplant systems in low- and middle-income countries.

This zone-based coordination model has enabled the deceased donor programme to remain operational despite significant infrastructural constraints. However, reliance on geographically bounded coordination in the absence of a fully executable national allocation system limits comprehensive oversight and complicates systematic monitoring of equity. In this context, centre-based and informal regional allocation practices may introduce variability in access and reduce transparency across the national programme.

## Governance and the implementation gap

Structured national audit mechanisms and registry-based monitoring have been shown to strengthen allocation transparency, support quality-improvement cycles, and enhance public trust in transplant governance (9).

Experience from emerging transplant systems suggests that sustainable deceased-donor programme expansion requires upfront and recurrent investment in coordination personnel, information infrastructure, laboratory capacity, and retrieval logistics to ensure safe and equitable allocation processes.

Implementation science literature in complex health systems indicates that policy adoption alone does not ensure programme performance unless accompanied by clearly defined operational accountability structures, protected coordination roles, and measurable system-level indicators (10, 11).

At the system level, governance limitations are more often related to diffuse operational accountability than to regulatory deficiency. Although statutory safeguards and Ministry of Health policies provide legitimacy and direction, day-to-day programme performance depends on clearly assigned responsibility for critical processes. These include donor identification, retrieval coordination, allocation documentation, immunobiology turnaround, and routine audit reporting. In the absence of protected roles, ring-fenced time, and explicit accountability mechanisms, programme implementation becomes vulnerable to variability arising from local resource constraints, individual commitment, and competing clinical service demands.

Addressing the implementation gap also requires recognition of the true cost structure of a functioning deceased donor programme. While deceased donor transplantation is frequently considered cost-saving when compared with long-term dialysis, safe national execution requires significant upfront and recurrent system investment. Essential components include dedicated transplant coordination personnel, secure information platforms for waitlisting and organ offer workflows, reliable and quality-assured immunobiology services with uninterrupted reagent supply and equipment maintenance, and efficient retrieval and transport logistics capable of meeting time-sensitive deceased donor pathways across geographic zones.

From a governance perspective, the absence of a mandatory national audit framework represents a particularly important limitation. Without systematic reporting of key performance indicators such as donor referral rates, consent conversion, organ offer timelines, crossmatch turnaround, cold ischaemia duration, and documented reasons for organ non-use policymakers are unable to distinguish between structural bottlenecks and centre-level performance variation. This also weakens the ability to justify targeted resource allocation. In this context, governance reform is closely linked to the development of information infrastructure. Establishing a minimum viable national transplant registry should thus be regarded not as an administrative enhancement, but as a central mechanism for accountability and programme learning.

## Allocation policy vs. real-world practice

In 2022, the Ministry of Health approved a national deceased-donor kidney allocation algorithm designed to provide a transparent and structured match-run process. This points-based framework represents an important transition from earlier centre-led or informal allocation practices, aiming to balance equity and clinical utility while maintaining immunological safety (8, 12, 19, 20).

The allocation model is based on a composite scoring system applied to ABO-compatible candidates on the national transplant waitlist. Recipient ranking incorporates multiple factors, including blood group compatibility, degree of HLA mismatch (penalised), sensitisation status (PRA/cPRA), accrued waiting time, donor–recipient age matching, and geographical proximity to minimise cold ischaemia time. Additional consideration is given to prior live donors. Priority tiers further refine allocation, with preferential weighting assigned to candidates with medical urgency, high sensitisation (PRA > 80%), and paediatric recipients—particularly in the context of younger donors. For each deceased donor offer, the highest-ranked candidates (typically the top 10) are shortlisted, and organs are sequentially allocated from highest to lowest score until acceptance. This structured framework is designed to promote transparency, reproducibility, and equitable access while accommodating immunological risk and logistical constraints.

However, the effective execution of this algorithm in routine practice depends on the consistent availability of critical operational infrastructure. In particular, three foundational enabling systems are required:

(i) a real-time national transplant waitlist with continuously updated candidate status;
(ii) timely and quality-assured immunobiology services capable of supporting compatibility assessment for every deceased-donor offer; and
(iii) a secure end-to-end organ offer and acceptance workflow that generates a permanent and auditable decision trail.

At present, these enabling systems are not yet uniformly established across the programme. As a result, attempting to implement fully algorithm-driven national match-runs in the absence of reliable data integrity, traceable workflows, and dependable immunobiology support may introduce avoidable risks to patient safety.

The specific operational gaps underlying these constraints, together with pragmatic system-level enablers required for progression, are summarised in Table 1.

**Table 1:** Key operational enablers required for consistent national allocation.

Domain	Current state/gap	Pragmatic enabler/next step
Waitlisting	Centre-held lists; national real-time visibility incomplete	Minimum viable national waitlist/registry with mandatory active/inactive status updates and minimum dataset (ABO, sensitisation history, centre)
Allocation	Zone-based coordination and manual allocation decisions	Standardised manual match-runs with mandatory documentation and equity metrics; phased transition to algorithmic match-runs as enabling systems mature
Offer workflow	Ad hoc communication; incomplete documentation of declines and timings	Secure offer-acceptance workflow capturing timestamps and reasons for declines; mandatory audit trail linked to the national registry
Immunobiology	Low-resolution or incomplete typing; limited rapid DSA/VXM availability; reliance on physical crossmatch	Phased strengthening with QA; defined turnaround times; scalable assays
Governance and accountability	Diffuse operational ownership and no ring-fenced implementation budget; inconsistent KPI reporting	Named national operational owners with ring-fenced implementation funding; mandatory KPI reporting and audit-feedback loops to donor hospitals and transplant centres
Donor identification	Limited ICU capacity; late referral of potential donors	Standardised referral triggers; ICU donor bundles; donor hospital recognition
Information systems	No real-time waitlist or donor registry; fragmented offer documentation	Minimum viable national registry; secure offer-acceptance platform; audit trail

DDKT, deceased donor kidney transplantation; KPI, key performance indicator; HLA, human leukocyte antigen; DSA, donor-specific antibody; VXM, virtual crossmatch; ABO, blood group compatibility; ICU, intensive care unit.

Consequently, allocation decisions continue to be largely coordinated within geographically defined retrieval networks aligned with the Colombo and Kandy zones. Within this operational context, transplant centres undertake documented manual allocation processes based on locally available clinical and immunological information. This pragmatic, safety-oriented interim approach has enabled the continued functioning of deceased-donor transplantation despite existing infrastructural limitations.

Nevertheless, reliance on manual and zone-restricted allocation introduces important system-level consequences. These include variability in the application of allocation criteria, reduced reproducibility of decision-making, limited national-level oversight, and constrained auditability. Such limitations have tangible implications, influencing perceptions of fairness among clinicians and patients while restricting the programme's ability to learn systematically from missed opportunities, allocation delays, and adverse outcomes.

Overall, the principal constraint lies not in the absence of policy direction but in the reliability of operational execution. Without dependable data systems, transparent decision pathways, and time-critical immunobiology support, a national allocation algorithm cannot function as intended. Until these foundational systems are strengthened, the allocation framework is likely to remain a well-defined policy construct rather than a consistently executable national process.

**Figure 2:**
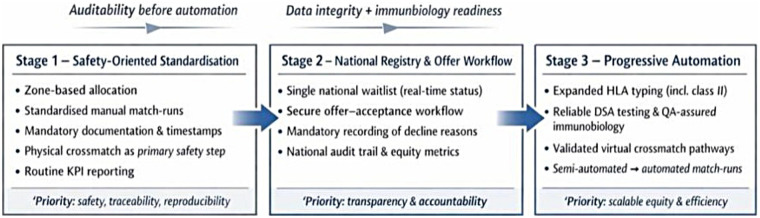
Staged roadmap for safe scale-up of deceased donor kidney transplantation in Sri Lanka. The figure illustrates a phased systems-implementation pathway emphasising progressive strengthening of allocation safety, traceability, and operational reliability before automation. DDKT, deceased donor kidney transplantation; KPI, key performance indicator; HLA, human leukocyte antigen; DSA, donor-specific antibody; VXM, virtual crossmatch.

As illustrated in Figure 2, the immediate programme priority is therefore to establish safety, traceability, and auditability before progressing towards incremental automation of allocation processes.

## Information systems as safety infrastructure

In well-established transplant systems, information platforms are sometimes viewed primarily as administrative tools. In an emerging deceased-donor programme, this perspective is misleading. In the Sri Lankan context, the absence of an integrated end-to-end transplant information system represents both a patient-safety concern and a governance vulnerability. In emerging transplant systems, incomplete data visibility can itself become a clinical risk factor, influencing allocation timing, compatibility assessment, and organ utilisation decisions.

The immediate requirement is not the development of a complex or resource-intensive digital platform. Rather, the priority is to establish a minimum viable national transplant registry that is secure, auditable, and mandatory for all transplant centres. At a basic level, such a registry should record candidate identifiers, demographic details, blood group, sensitisation and transplant history, centre affiliation, and real-time active or inactive waitlist status. In addition, it should capture every organ offer, document acceptance or decline decisions with reasons, record key time points across the allocation pathway, and track short-term clinical outcomes.

Even this minimum dataset would significantly strengthen programme governance. It would enable routine monitoring of key performance indicators, support structured audit and feedback mechanisms to donor hospitals and transplant centres, and provide defensible documentation of allocation fairness (9). Importantly, it would also support identification of recurrent delays or system failures within the donation-to-transplant pathway.

The absence of a reliable national data platform currently limits what the programme can safely achieve. Without accurate and accessible national information, it is difficult to conduct consistent allocation processes, monitor equity indicators, analyse causes of organ decline, or identify critical points of pathway failure such as donor maintenance challenges, retrieval delays, theatre access constraints, crossmatch turnaround time, transport logistics, or prolonged cold ischaemia. A transplant system that cannot systematically measure its own performance is unlikely to achieve sustained improvement and may struggle to maintain public confidence.

At present, Sri Lanka does not have a fully integrated national platform linking donor identification, consent processes, retrieval coordination, waitlisting, allocation decision-making, immunobiology assessment, offer acceptance documentation, and outcome tracking. Programme data are often maintained at individual centre level, and real-time national visibility remains limited. Continued reliance on manual communication methods—including telephone coordination and messaging-based decision pathways—reduces operational efficiency and weakens traceability. This, in turn, makes it more difficult to show that allocation decisions are consistently transparent, equitable, and clinically defensible.

## Immunobiology as a rate-limiting step

Immunobiology reliability currently represents the principal technical constraint limiting Sri Lanka's ability to conduct fully executable national match-runs for deceased-donor kidney transplantation. While policy frameworks often assume routine availability of donor-specific antibody (DSA) testing, virtual crossmatch (VXM), and high-resolution HLA typing, these services cannot yet be delivered consistently, rapidly, and at national scale in many emerging transplant systems (13, 14). This limitation is particularly relevant within the time-sensitive context of deceased donation. Similar immunogenetic service constraints have been described across several emerging transplant programmes, where laboratory turnaround reliability rather than conceptual protocol design represents the dominant barrier to safe national allocation.

A realistic strategy for programme development should thus focus on phased strengthening of reliability rather than early adoption of advanced or resource-intensive technologies. Immediate priorities include ensuring uninterrupted access to essential immunological assays, establishing defined turnaround times aligned with deceased-donor workflows, strengthening workforce training and retention, and implementing credible internal and external quality-assurance mechanisms. Only after these foundational elements are stabilised should the programme expand towards routine DSA-guided risk stratification, locally validated virtual crossmatch pathways, and gradually automated national allocation processes. In practical terms, the limiting factor is not conceptual protocol design but the dependable delivery of immunobiology services within deceased-donor time constraints.

Within this setting, physical crossmatching continues to represent an essential safety step in many deceased-donor allocation decisions, even when it introduces logistical delay or restricts broader organ sharing (15). This cautious approach is deliberate and clinically justified. Premature reliance on virtual crossmatching without robust local validation—particularly in populations that may be under-represented in commercial assay panels and reference datasets—risks false reassurance and avoidable immunological injury (16, 17). In a developing national programme, a single high-profile immunological complication linked to inadequate compatibility assessment could significantly undermine public and professional confidence.

In routine practice, compatibility assessment often depends primarily on ABO matching and available HLA typing data, which may be limited to low-resolution methods and incomplete characterisation of class II loci. Rapid and universally available DSA testing and validated virtual crossmatch services are not yet consistently achievable for every deceased-donor offer. Service continuity is further affected by workforce shortages, intermittent reagent availability, equipment maintenance challenges, and limited access to structured external quality-assurance programmes. These constraints are not merely technical inconveniences; they directly determine which allocation strategies can be implemented safely at national level.

## System bottlenecks limiting predictable allocation

Despite important progress in national policy development, several operational bottlenecks continue to limit the predictable functioning of deceased-donor kidney allocation in Sri Lanka. These challenges are largely foreseeable and arise across multiple stages of the donation-to-transplant pathway. Key constraints include limited dialysis capacity, variable effectiveness of donor identification and family consent processes, shortages of intensive care resources for donor maintenance, delays in theatre availability, logistical difficulties related to organ retrieval and transport, restricted crossmatch turnaround capacity, and the continued absence of an integrated national transplant information system (5, 7, 10, 11, 18).

Importantly, these bottlenecks rarely occur in isolation. Weakness at any single point in the pathway may lead to loss of potential donor opportunities, prolonged cold ischaemia time, or the need to make allocation decisions under suboptimal clinical or logistical conditions. The cumulative effect of interacting system limitations can thus reduce overall programme efficiency and increase the risk of avoidable inequity.

Within this setting, ethical safeguards and process traceability must be regarded as core design principles in accordance with internationally endorsed transplant governance frameworks (10, 11, 18) rather than optional administrative features.

These systemic challenges help explain why policy reforms alone have not yet translated into consistently consistent national allocation practice. The immediate priority for programme strengthening should thus focus on establishing minimum viable datasets, structured documentation pathways, and auditable workflows. Such measures would enable the transplant system to identify recurring weaknesses, standardise clinical practice across centres, and support safe and sustainable programme expansion.

## A staged roadmap for safe scale-up

Sri Lanka's deceased-donor kidney transplantation programme has now reached a developmental phase in which the principal requirement is not further policy articulation but clearer sequencing of implementation priorities. Building on the operational constraints outlined in the preceding section, a staged roadmap provides a pragmatic framework to translate policy intent into reproducible national practice. Rather than attempting immediate algorithm-driven automation, programme maturation is more likely to succeed through progressive stabilisation of safety-critical subsystems, including structured allocation documentation processes, reliable information-governance infrastructure, and time-sensitive immunobiology support. The proposed staged approach therefore reframes programme expansion as a forward-looking implementation pathway that prioritises system reliability and patient-safety safeguards as prerequisites for sustainable scale-up.

This staged implementation pathway can be operationalized through three sequential system level priorities.

(i) Standardised manual allocation processes supported by mandatory documentation and routine reporting of key performance indicators (KPIs),
(ii) Development of a minimum viable national transplant registry with a secure organ offer–acceptance workflow to enable traceability and monitoring of equity, and
(iii) Progressive automation of allocation processes supported by reliable and quality-assured immunobiology services.

This staged approach provides a realistic pathway for programme growth while maintaining patient safety, equitable access, and public trust.

### Stage 1: safety-oriented standardisation (immediate/foundational phase)

The initial stage focuses on strengthening patient safety, improving traceability, and enhancing reproducibility of allocation decisions in the absence of full national automation. During this phase, deceased-donor allocation continues to operate through geographically defined retrieval zones. However, manual allocation processes should be formally standardised.

All match-runs should be conducted using a nationally agreed structured proforma capturing donor characteristics, rationale for candidate selection, acceptance or decline decisions, and precise time points across the donation-to-transplant pathway. Importantly, manual allocation must be recognised as a governed interim system rather than an informal workaround. Each allocation decision should thus generate a permanent and reviewable audit record.

Routine monitoring of a core set of KPIs including time from donor referral to consent, organ offer-to-acceptance interval, crossmatch turnaround time, cold ischaemia duration, and documented reasons for organ non-use should be implemented at both zonal and national levels.

From an immunological perspective, this phase acknowledges current operational realities. Physical crossmatching remains the primary safety mechanism, and compatibility assessment is largely based on available ABO matching and low-resolution HLA typing data. This cautious strategy is intentional. Premature reliance on virtual crossmatch techniques without validated local immunobiology capacity may increase the risk of avoidable immunological complications and undermine programme credibility.

Successful completion of Stage 1 should be shown by consistent national use of standardised allocation documentation, routine KPI reporting, and clear traceability for every deceased-donor kidney offer.

### Stage 2: minimum viable national registry and offer workflow (short-term enabling phase)

The second stage introduces the essential information infrastructure required to support equitable national allocation. The objective is not to develop a complex digital platform, but to establish a secure, mandatory, and auditable national transplant registry.

This registry should maintain a unified national waitlist with real-time recording of active or inactive candidate status, basic immunological descriptors such as ABO group and sensitisation history, transplant centre affiliation, and unique patient identifiers. In parallel, a structured national organ offer-acceptance workflow should be implemented. Every deceased-donor kidney offer should be centrally logged, with automatic documentation of timing, acceptance or decline decisions, reasons for non-use, and short-term transplant outcomes.

Implementation of this stage would provide immediate governance benefits. It would enable monitoring of allocation equity, identification of systematic delays or disparities, and generation of defensible documentation demonstrating fairness of allocation decisions. Importantly, it would also support structured feedback to donor hospitals and transplant centres, enabling targeted quality-improvement initiatives rather than anecdotal or perception-driven reforms.

During Stage 2, allocation decisions may continue to involve manual or semi-manual processes. However, these decisions would occur within a transparent national framework, representing a transition from policy aspiration towards operational accountability.

Completion of this stage should be evidenced by national adoption of the registry platform, routine capture of organ offer-level data, and regular reporting of programme performance and equity indicators.

### Stage 3: progressive automation supported by quality-assured immunobiology (medium-term maturation phase)

The final stage involves cautious introduction of automated allocation processes once reliable data infrastructure and service continuity have been established. Programme development during this phase should prioritise strengthening immunobiology capacity in parallel with gradual automation. Key priorities include expansion and standardisation of HLA typing particularly for class II loci reliable access to donor-specific antibody testing, clearly defined laboratory turnaround targets aligned with deceased-donor timelines, and robust participation in internal and external quality-assurance systems.

Where local validation is achieved, virtual crossmatch approaches may be introduced incrementally to support broader sharing and reduce cold ischaemia time. Nevertheless, physical crossmatching should remain available as an essential safety safeguard. Early automation should support clinical decision-making by generating ranked candidate lists while preserving the ability for clinicians to document justified deviations based on contextual factors.

At this stage, the national allocation algorithm can begin to function as originally intended—as a consistent, points-based system balancing equity and clinical utility, supported by reliable data systems and strengthened immunological safety. Automation should thus be understood as the outcome of successful system strengthening rather than its starting point.

Progression beyond Stage 3 should be supported by demonstrated improvements in immunobiology turnaround performance, successful pilot implementation of automated match-runs with confirmatory audit review, and measurable reduction in allocation variability without compromise to patient safety.

### Why the staged approach is important?

This structured roadmap deliberately avoids a common challenge observed in many LMIC transplant programmes: premature adoption of high-income-country allocation models without adequate supporting infrastructure. By prioritising safety and accountability before technological automation, the proposed pathway reduces the risk of inequitable allocation, preventable immunological complications, and erosion of public confidence (10, 11, 18, 21). The approach may thus offer relevant lessons for other resource-constrained transplant systems facing similar implementation realities.

## Discussion

Sri Lanka's experience reflects a common transition point seen in many low- and middle-income country transplant programmes. Once policy frameworks and basic deceased-donor retrieval pathways are established, the principal constraint progressively shifts towards the strength of implementation infrastructure that enables allocation decisions to be consistent and auditable in routine clinical practice.

In this context, the proposed staged roadmap emphasises the importance of strengthening safety mechanisms before pursuing technological automation. Priority actions include the standardisation of manual allocation processes with structured documentation and routine KPI reporting, establishment of a minimum viable national transplant registry with a secure organ offer workflow, and phased development of reliable, time-critical immunobiology services supported by credible quality-assurance systems.

This systems-oriented approach highlights that equitable allocation depends not only on the design of a national algorithm, but also on the reliable functioning of underlying processes. Data completeness, laboratory turnaround performance, and the ability to review each deceased-donor opportunity in a transparent manner are central determinants of programme credibility.

While the operational constraints and health system architecture described are specific to Sri Lanka, the underlying principles of a staged, safety-first approach to deceased donor kidney allocation are broadly applicable across low- and middle-income countries. In particular, the emphasis on establishing foundational elements—such as a reliable and auditable waitlist, transparent offer–acceptance pathways, and quality-assured immunobiological support—prior to full algorithmic allocation may provide a pragmatic template for other emerging transplant systems. Rather than direct replication, this framework is intended to be adaptable, allowing individual countries to develop context-sensitive, stepwise implementation strategies aligned with their own infrastructural, financial, and workforce realities (22). Such an approach may help balance equity, safety, and scalability while avoiding premature automation in the absence of critical enabling systems.

By analysing current bottlenecks within a staged and safety-focused implementation framework, this Perspective provides a pragmatic strategy for strengthening deceased-donor transplantation capacity in Sri Lanka. The lessons outlined may also be relevant to other LMIC transplant programmes seeking to expand access to transplantation while maintaining patient safety, equitable allocation, and sustained public trust.

## Limitations

This Perspective has several limitations. Sri Lanka currently lacks a fully operational national transplant registry with harmonised routine reporting across centres. As a result, some system-level descriptions rely on aggregated estimates and centre-held records rather than a single verifiable national dataset. Descriptive programme statistics presented in this Perspective represent system-level estimates derived from centre-reported activity summaries and professional consensus observations, as a fully harmonised national transplant registry dataset is not yet operational.

The article does not present new primary data, qualitative interviews, or a structured national audit of organ offer processes, cold ischaemia times, or clinical outcomes. Consequently, it is not possible to quantify the relative contribution of individual bottlenecks such as intensive care capacity constraints, theatre access delays, crossmatch turnaround limitations, or transport logistics to missed donor opportunities.

In addition, policy frameworks and operational workflows in Sri Lanka continue to evolve. Some implementation details described in this Perspective may thus change over time. The proposed staged roadmap should be interpreted as a pragmatic systems-level framework rather than a predictive model of programme impact.

Finally, caution is required when considering transferability of these recommendations to other LMIC settings. Differences in health-system organisation, resource availability, regulatory environments, and transplant epidemiology may influence both sequencing and feasibility of implementation strategies.

## Conclusion

Safe expansion of deceased-donor kidney transplantation in resource-limited settings is determined less by policy ambition alone than by the gradual strengthening of implementation systems that ensure transparency, traceability, and immunological safety in routine clinical practice. Sri Lanka's evolving experience illustrates that sustainable programme growth depends on strengthening the operational foundations of allocation, including structured documentation, reliable information infrastructure, and time-critical immunobiology capacity, before pursuing technological automation. Framing national transplant development as a staged systems-engineering process rather than a purely clinical or legislative endeavour provides a pragmatic pathway towards equitable access, consistent decision-making, and durable public trust. This implementation-focused perspective may offer a transferable conceptual model for other low- and middle-income countries seeking to expand transplantation services while safeguarding safety, fairness, and long-term programme legitimacy (10, 11).

## References

[1] Wolfe RA Ashby VB Milford EL Ojo AO Ettenger RE Agodoa LYC Comparison of mortality in all patients on dialysis, patients on dialysis awaiting transplantation, and recipients of a first cadaveric transplant. N Engl J Med. (1999) 341(23):1725–30. 10.1056/NEJM199912023412303 10580071

[2] Tonelli M Wiebe N Knoll G Bello A Browne S Jadhav D Systematic review: kidney transplantation compared with dialysis in clinically relevant outcomes. Am J Transplant. (2011) 11(10):2093–109. 10.1111/j.1600-6143.2011.03686.x 21883901

[3] Meier-Kriesche HU Kaplan B. Waiting time on dialysis as the strongest modifiable risk factor for renal transplant outcomes. Transplantation. (2002) 74(10):1377–81. 10.1097/00007890-200211270-00005 12451234

[4] Chadban SJ Ahn C Axelrod DA Foster BJ Kasiske BL Kher V KDIGO clinical practice guideline on the evaluation and management of candidates for kidney transplantation. Transplantation. (2020) 104(4 Suppl 1):S11–103. 10.1097/TP.0000000000003136 32301874

[5] Liyanage T Ninomiya T Jha V Neal B Patrice HM Okpechi I Worldwide access to treatment for end-stage kidney disease: a systematic review. Lancet. (2015) 385(9981):1975–82. 10.1016/S0140-6736(14)61601-9 25777665

[6] Kramer A Pippias M Noordzij M Stel VS Afentakis N Ambühl PM The ERA-EDTA registry annual report: a summary. Clin Kidney J. (2018) 11(1):108–22. 10.1093/ckj/sfx149 29423210 PMC5798130

[7] Global Observatory on Donation and Transplantation (GODT). Global report on donation and transplantation: 2024 data. Coordinated by WHO-ONT; (2025). Available online at: https://www.transplant-observatory.org/wp-content/uploads/2025/12/2024-data-global-report.pdf (Accessed February 8, 2026).

[8] Ministry of Health, Sri Lanka. National policy on organ, tissue and cell transplantation of Sri Lanka 2021–2030. Colombo: Ministry of Health; (2022). Available online at: https://www.health.gov.lk/wp-content/uploads/2022/10/10-National-Policy-on-Organ-Tissue-and-Cell-Transplantation-of-Sri-Lanka-2021-2030-compressed.pdf (Accessed February 8, 2026).

[9] Tsapepas D King KL Husain SA Mohan S. Evaluation of kidney allocation critical data validity in the OPTN registry using dialysis dates. Am J Transplant. (2020) 20(1):318–9. 10.1111/ajt.15616 31550418

[10] World Health Organization. WHO guiding principles on human cell, tissue and organ transplantation. Geneva: World Health Organization; (2010). Available online at: https://apps.who.int/iris/handle/10665/341814 (Accessed February 8, 2026).

[11] Delmonico FL Domínguez-Gil B Matesanz R Noel L. A call for government accountability to achieve national self-sufficiency in organ donation and transplantation. Lancet. (2011) 378(9800):1414–8. 10.1016/S0140-6736(11)61486-4 22000137

[12] Yeung MY Coates PT Li PKT. Kidney organ allocation system: how to be fair. Semin Nephrol. (2022) 42(4):151274. 10.1016/j.semnephrol.2022.09.002 36566139

[13] Stegall MD Smith BH Bentall AJ Dean PG Diwan T Gloor JM. Sensitization in transplantation: assessment of risk and approaches to management. Clin Transplant. (2016) 30(12):1606–17.

[14] Tait BD Süsal C Gebel HM Nickerson PW Zachary AA Claas FHJ Consensus guidelines on the testing and clinical management issues associated with HLA and non-HLA antibodies in transplantation. Transplantation. (2013) 95(1):19–47. 10.1097/TP.0b013e31827a19cc 23238534

[15] Peacock S Briggs D Barnardo M Battle R Brookes P Callaghan C BSHI/BTS guidance on crossmatching before deceased donor kidney transplantation. Int J Immunogenet. (2022) 49(1):22–9. 10.1111/iji.12558 34555264 PMC9292213

[16] Bhaskaran MC Heidt S Muthukumar T. Principles of virtual crossmatch testing for kidney transplantation. Kidney Int Rep. (2022) 7(6):1179–88. 10.1016/j.ekir.2022.03.006 35685330 PMC9171621

[17] Lefaucheur C Louis K Morris AB Taupin JL Nickerson P Tambur AR Clinical recommendations for posttransplant assessment of anti-HLA donor-specific antibodies: a sensitization in transplantation: assessment of risk (STAR) consensus document. Am J Transplant. (2023) 23(1):115–32. 10.1016/j.ajt.2022.11.013 36695614

[18] World Health Assembly. Increasing availability, ethical access and oversight of transplantation of human cells, tissues and organs. Resolution WHA77.4. Geneva: World Health Organization; (2024). Available online at: https://apps.who.int/gb/ebwha/pdf_files/WHA77/A77_R4-en.pdf (Accessed February 8, 2026).

[19] Organ Procurement and Transplantation Network (OPTN). OPTN policies: policy 8—allocation of kidneys (KAS policy & guidance). Rockville (MD): Health Resources and Services Administration (HRSA) (2025). Available online at: https://www.hrsa.gov/optn/professionals/resources/kidney-pancreas/kidney-allocation-system (Accessed February 8, 2026).

[20] Persijn GG Gabeler EE van der Woude FJ. Matching and allocation in kidney transplantation: lessons from eurotransplant. Kidney Int Suppl. (2005) 94:S7–12. 10.1111/j.1523-1755.2005.09402.x

[21] Moazam F Zaman RM Jafarey AM. Conversations with kidney vendors in Pakistan: an ethnographic study. Hastings Cent Rep. (2009) 39(3):29–44. 10.1353/hcr.0.0136 19537621

[22] Reese PP Caplan AL Bloom RD Abt PL. How should we use kidney paired donation to increase transplantation? Clin J Am Soc Nephrol. (2015) 10(10):1880–8. 10.2215/CJN.00030115

